# c-Met signaling promotes IL-6-induced myeloma cell proliferation

**DOI:** 10.1111/j.1600-0609.2009.01212.x

**Published:** 2009-04

**Authors:** Håkon Hov, Erming Tian, Toril Holien, Randi Utne Holt, Thea K Våtsveen, Unn-Merete Fagerli, Anders Waage, Magne Børset, Anders Sundan

**Affiliations:** 1 Department of Cancer Research and Molecular Medicine, Norwegian University of Science and TechnologyTrondheim; 2 Department of Oncoclogy, St Olav University HospitalTrondheim; 3 Section of Hematology, St Olav University HospitalTrondheim, Norway

**Keywords:** c-Met, multiple myeloma, receptor tyrosine kinase, proliferation, intracellular signaling

## Abstract

**Objectives:**

Hepatocyte growth factor (HGF) is a constituent of the myeloma microenvironment and is elevated in sera from myeloma patients compared to healthy individuals. Increased levels of serum HGF predict a poor prognosis. It has previously been shown by us and others HGF can act as a growth factor to myeloma cells *in vitro* although these effects have been moderate. We therefore wanted to investigate if HGF could influence the effects of interleukin (IL)-6.

**Methods:**

Myeloma cell lines and primary samples were tested for the combined effects of IL-6 and HGF in inducing DNA synthesis and migration. Expression levels of c-Met protein were analysed by Western blotting and flow cytometry. Signaling pathways were examined by Western blotting using phosphospecific antibodies and a Ras-GTP pull down assay.

**Results:**

HGF potentiated IL-6-induced growth in human myeloma cell lines and in purified primary myeloma cells. There was also cooperation between HGF and IL-6 in induction of migration. There seemed to be two explanations for this synergy. IL-6-treatment increased the expression of c-Met making cells HGF responsive, and IL-6 was dependent on c-Met signaling in activating both Ras and p44/42 MAPK by a mechanism involving the tyrosine phosphatase Shp2.

**Conclusions:**

The results indicate that besides from being a myeloma growth factor alone, HGF can also potentiate the effects of IL-6 in myeloma proliferation and migration. Thus, c-Met signaling could be a target for therapy of multiple myeloma.

Identification of signaling pathways in tumor cells able to promote growth and survival of the malignant cells are important for targeted treatment of cancers. Multiple myeloma is a cancer caused by clonal expansion of malignant plasma cells that are usually confined to the bone marrow. Isolated primary myeloma cells only rarely grow or survive outside of the bone marrow microenvironment. A number of growth and anti-apoptotic factors, including interleukin-6 (IL-6), have been implicated in sustaining the malignant myeloma cells (1).

Some 10 yr ago, we found that hepatocyte growth factor (HGF) may play a role in multiple myeloma, a finding later confirmed by various techniques in different laboratories. The main results were that myeloma cells produce HGF (2, 3), and that high serum levels of HGF at diagnosis correlated with poor prognosis for patients (4). Compared to healthy controls, bone marrow plasma from multiple myeloma patients contained high levels of HGF (5). However, also in healthy persons, HGF could be detected, both in bone marrow plasma and serum. It has previously been shown by us and others that myeloma cells express the HGF-receptor c-Met (2, 3, 6, 7).

Recently, HGF and c-Met have been found to be significantly dysregulated in gene expression profiling experiments on purified plasma cells from multiple myeloma patients. HGF was the only growth factor among 70 highly expressed genes in malignant plasma cells compared to normal bone marrow plasma cells (8), and HGF and IL-6 were also shown to characterize one of four clusters of hyperdiploid myeloma (9). Furthermore, in a study comparing transcriptional signatures between cells from patients with multiple myeloma, chronic lymphocytic leukaemia, and Waldenströms macroglobulinaemia, both HGF and *MET* as well as the receptor for IL-6, were on the list of genes distinguishing myeloma from the latter two conditions (10). Despite these findings, HGF generally appears to be a weak growth factor for myeloma cells *in vitro*. Though there are exceptions (6, 11, 12), when tested for ability to induce cell proliferation or prevent apoptosis in a large number of myeloma cell lines or primary myeloma cells, HGF generally have had limited effects (H. Hov and M. Børset, unpublished data).

*MET* was first cloned as a transforming gene from a chemically transformed osteosarcoma cell line (13), later HGF was identified as the only known ligand for c-Met (14). c-Met signaling is essential for fetal development, wound healing, and tissue regeneration in the adult organism (15–20). Aberrant c-Met signaling has been implicated in a large number of tumors (21, 22). The receptor has been suggested to be important in creating or maintaining a more malignant phenotype (23). c-Met tyrosine kinase activation initiates complex downstream signaling cascades involving several intracellular signaling pathways. Such signaling pathways may however, be shared by several receptor tyrosine kinases, and substantial crosstalk may exist between signaling pathways downstream of diverse receptors. Thus, under certain circumstances, the signal from one receptor tyrosine kinase may be replaced with the signal from another receptor, or the signals from two receptor kinases may act in concert and potentiate each other.

Here, we present data indicating that c-Met signaling promotes growth-stimulatory signaling from IL-6. Thus, in myeloma cells, the presence of c-Met signaling may be necessary to obtain full effect of other growth factors. Conversely, IL-6 is also necessary to obtain full effect of HGF in cell migration by increasing expression of HGF’s receptor c-Met. The results suggest that targeting c-Met signaling may attenuate cell proliferation induced by other growth factors such as IL-6, and may therefore represent a novel approach to cancer treatment also in cancers that at first sight seem independent of c-Met signaling.

## Materials and methods

### Reagents

Recombinant human IL-6 was from R&D Systems (Abingdon, UK). HGF was purified from the human myeloma cell line JJN-3 as described previously (3) or purchased from PeproTech EC Ltd (London, UK). The c-Met tyrosine kinase inhibitor PHA-665752 (24) was a kind gift from J. G. Christensen (Pfizer Inc., New York, NY, USA). The Shp2 inhibitor NSC-87877 and the MEK1/2 inhibitors PD98059 and U126 were from Merck Chemicals Ltd (Nottingham, UK). The following c-Met antibodies were used: clone DL-21 from Upstate (Waltham, MA, USA); Met (25H2) and anti-phospho-Tyr^1349^c-Met from Cell Signaling Technology (Beverly, MA, USA); Fluorescein isothiocyanate (FITC) labeled anti-human c-Met, eBioclone 97, from eBioscience (San Diego, CA, USA); the neutralizing antibody clone 95309 from R&D Systems. Anti-Shp2, anti-phospho-Tyr^542^Shp2, anti-phospho-Tyr^580^Shp2, and anti-Gab1 were from Upstate (Lake Placid, NY, USA). Anti-phospho-Ser^473^Akt, anti-phospho-Tyr^705^STAT3, anti-STAT3, anti-phospho-Thr^202^/phospho-Tyr^204^-p44/42 MAPK, anti-p44/42 MAPK, anti-phospho-Tyr^307^Gab1, and anti-phospho-Tyr^627^Gab1 were from Cell Signaling Technology. Anti-GAPDH was from Abcam (Cambridge, UK). Rabbit anti-HGF serum was raised by us as previously described (4).

### Cell lines and primary patient samples

ANBL-6 cells and INA-6 cells were kind gifts from Dr Diane Jelinek (Mayo Clinic, Rochester, MN, USA) and Dr Martin Gramatzki (University of Erlangen-Nuremberg, Erlangen, Germany), respectively. OH-2 and IH-1 were established in our laboratory as described previously (25, 26). Cell lines were grown in RPMI 1640 with 10% fetal calf serum (FCS) or human serum (OH-2 and IH-1), 2 mmol/L l-glutamine, and 40 μg/mL gentamicin (complete medium) and 1 ng/mL IL-6.

CD138-positive cells were purified from left over material from bone marrow aspirates taken for diagnostic purposes by immunomagnetic separation (27). Myeloma cells were purified using Macs MicroBeads (Miltenyi Biotec, Auburn, CA, USA). The use of bone marrow aspirates for this purpose was approved by the regional ethics committee and by informed consent from the patients.

### Proliferation assay

Cells were washed four times in Hank’s balanced salt solution (HBSS), seeded in 96-well plastic culture plates (Corning Costar, Corning, NY, USA) at 1–10 × 10^4^ cells/well in 200 μL of 0.1% bovine serum albumin (BSA) (cell lines) or 1% FCS (primary cells) in RPMI 1640 with 2 mmol/L l-glutamine, and 40 μg/mL gentamicin (serum-free media). After 48 h 1 μCi of methyl-[^3^H]thymidine (NEN Life Science Products, Boston, MA, USA) was added per well and cells were harvested either 6 or 18 h later with a Micromate 96-well harvester (Packard, Meriden, CT, USA). ß-radiation was measured with a Matrix 96 ß counter (Packard).

### Migration assay

INA-6 cells were washed four times in HBSS, resuspended in serum-free media, and seeded (2 × 10^6^ cells/well) in the top compartments of polycarbonate transwells (pore size, 5 μm; Corning Costar). The total volume was 100 μL in the top compartments and 600 μL in the bottom compartment. All samples were performed in duplicates. After 18 h, the number of cells that had migrated through the membrane to the bottom chamber was determined by a Coulter Counter Z1 (Beckman Coulter, Fullerton, CA, USA).

### Immunoblotting

Cells were washed four times in HBSS and seeded at 10^6^ cells/mL in serum-free media with or without cytokines. PHA-665752 was added 15–30 min prior to cytokines. To detect phosphorylated Gab1, Shp2, and c-Met in ANBL-6, cells were depleted of FCS and IL-6 by four washes in HBSS, and seeded at 10^6^ cells/mL in RPMI 1640 with 0.1% BSA and a 1 : 750 dilution of rabbit anti-HGF serum over night. Cells were then washed four times in HBSS and seeded in 0.25 mL of RPMI 1640 with 0.1% BSA in 24-well plates (4 × 10^6^ cells/well). PHA-665752 was added to the wells (were indicated) 15 min before incubation with HGF or IL-6 for 10 min. Then, cells were counted by a Coulter Counter Z1 (Beckman Coulter, Fullerton, CA, USA), pelleted, and resuspended in 20 μL lysis buffer (11) per 500 000 cells. Thereafter, immunoblotting was performed as previously described (11).

### Flow cytometry

Cells were washed four times in HBSS and seeded at a concentration of 250 000/mL in serum-free media. After overnight incubation with cytokines, cells were labeled with 0.25 μg FITC-conjugated anti-c-Met antibody (eBioscience, San Diego, CA, USA. Catalog number 11-8858) or 0.25 μg FITC-conjugated isotype control antibody (Cat. no. 11-4301). Viable cells were gated from the forward-, side-scatter dot plot, and analyzed for fluorescence.

### Ras activation assay

Ras activation was measured with a Ras activation kit (Stressgen Bioreagents, Victoria, BC, Canada) according to the manufacturer’s protocol. Briefly, ANBL-6 cells were washed four times in HBSS and serum starved for 4 h, incubated with 200 nm PHA-665752 for 30 min, and then stimulated with cytokines for another 10 min. Cells were pelleted and lysed in buffer containing Complete Mini protease inhibitor tablets (Roche, Basel, Switzerland). Lysates from 6 × 10^6^ cells were incubated with 80 μg of a Glutathion *S*-transferase fusion protein containing the Ras binding domain of Raf1. Lysates were thereafter placed on an immobilized glutathione disc on a spin column for 1 h at 4°C with gentle rocking. The columns were washed and eluted with 50 μL SDS sample buffer containing β-mercaptoethanol. Twenty-five microlitre of sample were subjected to gel electrophoresis and Western blotting, and membranes were probed with a specific Ras antibody. Unfractionated lysates were similarly subjected to immunoblotting to control total amount of Ras.

### Fluorescent *in situ* hybridization analysis

Cytospin slides were used for fluorescent *in situ* hybridization (FISH) analysis. Hybridization was performed using standard procedure (Vysis, Downers Grove, IL, USA). Thereafter, cells were counterstained with DAPI (Vysis) and scored using a Nikon Eclipse 90i epifluorescence microscope with PlanApo VC 60x/1.4oel (Nikon Instruments Europe, Badhoevedorp, the Netherlands), and software CytoVision version 3.7 Build 58, 2005 (Applied Imaging, San Jose, CA, USA). Information on probes is available in a Table S1.

### Statistics

The statistical significance was determined using two-tailed, unpaired Student’s *t*-test. The minimal level of significance was *P*= 0.05.

## Results

### IL-6 augmented HGF-effects by increasing c-Met expression

**Figure 1:**
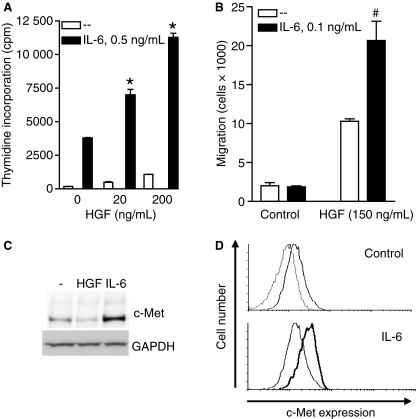
Synergistic effects between HGF and IL-6 in proliferation and migration of INA-6 cells. (A) INA-6 cells were grown in serum-free media with IL-6 and HGF as indicated for 3 d before estimation of DNA synthesis. Error bars represent SEM of triplicate measurements. * Denotes statistically significant difference from the IL-6 alone situation (*P*< 0.05). (B) INA-6 cells were seeded in the top wells of transwell migration chambers. HGF was added to the bottom wells and IL-6 to both top and bottom wells. After 18 h, migration was determined as described in Materials and methods. Error bars represent SEM of duplicate measurements. #Denotes statistical significant difference between HGF with or without IL-6 was not reached (*P*-value = 0.14). (C) INA-6 cells were grown in serum-free media with or without 100 ng/mL HGF or 1 ng/mL IL-6 over night, then harvested, lysed, and subjected to gel electrophoresis and Western blotting. The membrane was probed with an anti-c-Met antibody and a GAPDH antibody as loading control. (D) INA-6 cells were grown in serum-free media with or without 1 ng/mL IL-6 over night, labeled with FITC-conjugated antibody against c-Met or isotype control antibody and subjected to flow cytometry analysis. Upper panel – untreated cells labeled with FITC-c-Met antibody (bold line) compared with isotype control antibody (thin line); lower panel – c-Met expression in IL-6 treated cells (bold line) compared to untreated cells (thin line).

Even though HGF activates c-Met in INA-6 cells (11) the effects of HGF on cell proliferation in this cell line are moderate. Thus, in the absence of other growth factors, HGF-induced proliferation was limited (Fig. 1A). Interestingly, the presence of HGF together with IL-6 potentiated cell proliferation compared to the proliferation obtained with IL-6 alone (Fig. 1A). HGF had stronger effects in migration of INA-6 cells (28) (Fig. 1B), while there was no migration after IL-6 treatment. However, IL-6 increased migration by HGF substantially.

**Figure 2:**
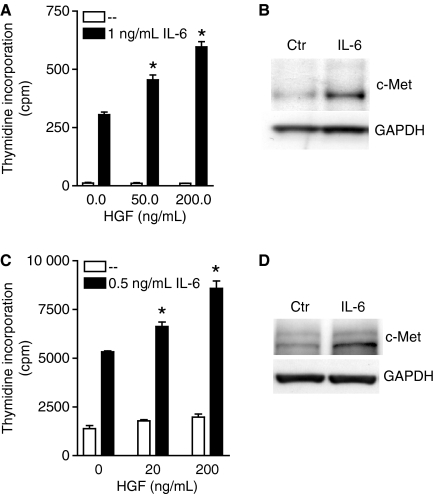
HGF potentiated IL-6-induced proliferation in OH-2 and IH-1 cells. OH-2 (A) and IH-1 (C) cells were grown in serum-free media with or without IL-6 and HGF as indicated for 3 d before estimation of DNA synthesis. Error bars represent SEM of triplicate measurements. OH-2 (B) and IH-1 (D) were grown in serum-free media with or without 1 ng/mL IL-6 over night, then harvested, lysed, and subjected to gel electrophoresis and Western blotting. The membrane was probed with an anti-c-Met antibody and a GAPDH antibody as loading control. * Denotes statistically significant difference from the IL-6 alone situation (*P*< 0.05).

A simple explanation for these findings could be that HGF receptor expression was low and rate limiting for HGF signaling. Indeed, after 20-h treatment with IL-6 the expression of c-Met protein in INA-6 was elevated compared to the expression in untreated cells (Fig. 1C). The presence of HGF downregulated c-Met expression as this study and many other studies also have shown previously (29). Similar results were obtained when c-Met cell surface expression was analyzed by flow cytometry. Cells treated with IL-6 (Fig. 1D lower panel) had higher surface expression of c-Met than untreated cells (Fig. 1D upper panel). Also in the myeloma cell lines OH-2 (Fig. 2A,B) and IH-1 (Fig. 2C,D) similar results were seen: HGF alone did not increase proliferation but potentiated the effect of IL-6, and likewise, incubation with IL-6 increased the expression of c-Met.

### Inhibition of c-Met signaling reduced IL-6-induced proliferation

**Figure 3:**
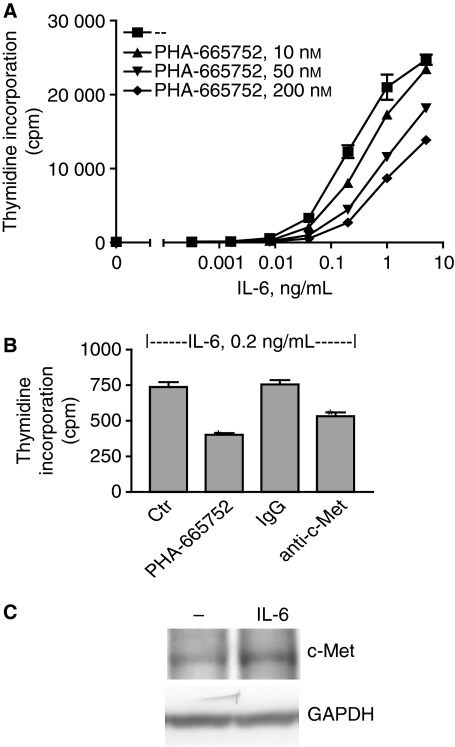
Inhibition of c-Met signaling reduced IL-6-induced proliferation in ANBL-6 cells. (A and B) ANBL-6 cells were grown in serum-free media for 3 d with combinations of different concentrations of IL-6 and the c-Met tyrosine kinase inhibitor PHA-665752 or 10 μg/mL of an antibody blocking HGF binding to c-Met or 10 μg/mL of a control antibody of the same isotype before estimation of DNA synthesis. The concentration of PHA-665752 in B was 200 nm. Error bars represent SEM of triplicate measurements. In (A) incorporation of thymidine at 50 and 100 nm PHA-665752 were statistically significant different from the control at IL-6 concentrations above 0.1 ng/mL. In (B) * denotes statistically significant difference from the control or IgG-treatment (*P*< 0.05). (C) ANBL-6 cells were grown in serum-free media with or without 1 ng/mL IL-6 over night. Immunoblots were probed with antibodies against c-Met and GAPDH.

We have previously demonstrated an autocrine HGF-c-Met loop promoting growth of the myeloma cell line ANBL-6 (11). However, under serum-free conditions there was almost no baseline proliferation in ANBL-6 cells, suggesting that the HGF-c-Met loop could not sustain proliferation on its own (Fig. 3A). IL-6 promoted growth of the cells in a dose-dependent manner. Surprisingly, inhibiting c-Met signaling with the specific c-Met tyrosine kinase inhibitor, PHA-665752, in the presence of IL-6 gave a potent and dose-dependent reduction in cell proliferation (Fig. 3A). To confirm that c-Met activation was important for IL-6-induced proliferation, the kinase inhibitor was replaced by an antibody blocking HGF binding to c-Met (Fig. 3B). The antibody reduced IL-6-induced proliferation to a similar extent as did the c-Met kinase inhibitor. Taken together, the results indicate that IL-6 is dependent on c-Met signaling for full growth promotion also in the ANBL-6 cell line. However, there were no clear differences in c-Met expression after IL-6 treatment in these cells (Fig. 3C), indicating that some other mechanism than receptor upregulation is responsible for the dependency on c-Met signaling in IL-6-induced proliferation.

### IL-6-induced proliferation was dependent on activated c-Met in some primary myeloma cells

**Figure 4:**
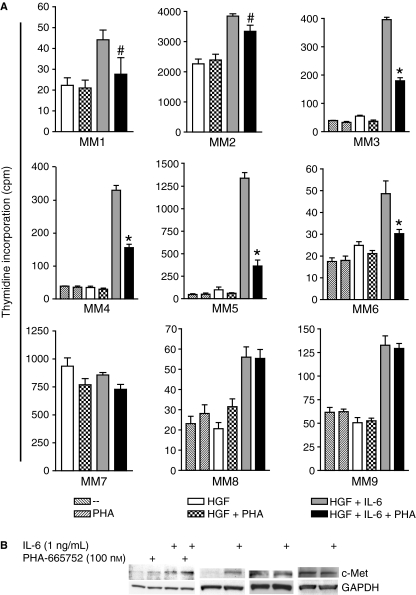
Inhibition of c-Met by PHA-665752 reduced the effects of IL-6-induced proliferation in purified primary myeloma cells. (A) Purified primary myeloma cells were grown with or without IL-6 or 50 ng/mL HGF in combination with 100 nm PHA-665752 (PHA). The concentrations of IL-6 were 0.1 ng/mL for MM2, 0.5 ng/mL for MM8 and 1 ng/mL for the rest. DNA synthesis was estimated after 2 or 3 d. Error bars represent SEM of triplicate measurements. #The difference in thymidine incorporation between IL-6 with or without PHA-665752 did not reach statistic significance as *P*-values were 0.13 and 0.08 for MM1 and MM2 respectively. *Denotes statistically significant difference from IL-6 without PHA-665752 (*P*< 0.05). (B) Western blots of cells from the patients MM2, 4, 3, and 9 are presented from left to right. Cells were treated the same way as in the thymidine incorporation assays except that serum was omitted and cells lysed after 18 h of incubation. The level of c-Met expression was determined by Western blotting. An anti-GAPDH antibody was used as loading control.

We found nine primary isolates out of 12 tested that responded reasonably well to IL-6 in the presence of HGF. As often is the case with primary myeloma samples, the DNA synthesis between samples showed considerable variation. Inhibiting c-Met with PHA-665752 reduced IL-6-induced proliferation in six samples (Fig. 4A, MM1–MM6); however, in two of the samples the changes were minor (MM1 and MM2). These results suggest that c-Met signaling is required for full effect of IL-6 also in some primary myeloma cells. In two of the samples (MM8 and MM9), IL-6-induced proliferation was not affected by the presence of the c-Met inhibitor. IL-6 can therefore also promote cell proliferation independently of c-Met. The expression of c-Met was only examined in four of the patients because of limited amounts of cells (Fig. 4B). The level of c-Met was low in untreated cells but increased with IL-6 in the patient samples MM2 and MM4, which is similar to the results obtained with the INA-6, OH-2, and IH-1 cell lines. There seemed to be no increase in c-Met expression after IL-6 stimulation in the patient sample MM3 despite dependence on c-Met in IL-6-induced proliferation in these cells. This is similar to findings in the ANBL-6 cell line suggesting other mechanisms for synergy between IL-6 and HGF than IL-6-induced upregulation of c-Met expression. In the patient sample MM9, the IL-6-induced proliferation was not dependent on c-Met signaling, and there was no increase of c-Met expression after IL-6 treatment (Fig. 4A,B). Because elevated HGF expression has been reported to characterize a subgroup of the hyperdiploid myeloma patients, we analyzed some of the most common genetic aberrations in our primary samples by FISH (Table 1 and Materials and methods). Of the responders, two had IgH translocations while one had not. Response to c-Met inhibition was therefore not dependent on the presence or absence of an IgH translocation. None of the non-responding patients was positive for IgH tranlocations.

**Table 1:** Genetic aberrations in patient samples

Sample	IgH split	t(11;14)	t(4;14)	del13	del17	t(6;14)	t(14;16)	Sample taken
MM1	1	0	0	0	0	1	0	D
MM2								R
MM3								R
MM4	1	1	0	0	0			D
MM5	0			0	0			D
MM6								D
MM7	0			0	0			D
MM8	0			0	0			D
MM9	0		0	1	0			D

Empty cells, not determined; 1, present; 0, absent; D, at diagnosis; R, at relapse.

### IL-6-activation of Ras-MAPK signaling was c-Met dependent

**Figure 5:**
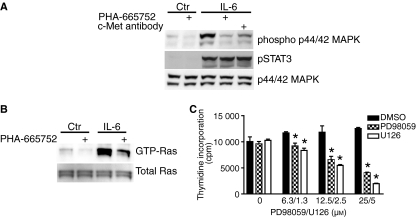
c-Met signaling was necessary for IL-6-induced activation of the Ras-MAPK pathway in ANBL-6 cells. (A) ANBL-6 cells were washed and serum starved for 4 h allowing endogenous HGF-production, preincubated with 200 nm PHA-665752 or 2 μg/mL of an antibody blocking HGF binding to c-Met for 30 min and stimulated with 1 ng/mL IL-6 for 10 min. Cells were lysed and subjected to Western blotting. Membranes were probed with antibodies against phospho-p44/42 MAPK or phospho-STAT3 tyr 705, stripped and reprobed with an antibody against total p44/42 MAPK. (B) Lysates from ANBL-6 were made as in (A) and subjected to affinity precipitation with a Raf1-Ras binding domain-GST fusion protein to pull down GTP-bound Ras and then detected on Western blot with a Ras antibody. Total Ras was detected in the same lysates to ensure equal amounts of Ras in the experiment (lower panel). (C) ANBL-6 cells were grown in serum-free media in the presence of 0.1 ng/mL IL-6 with or without the MEK1/2 inhibitors PD98059 or U126 for 3 d before estimation of DNA synthesis. Error bars represent SEM of triplicate measurements. * Denotes statistically significant difference from the situation without inhibitors (*P*< 0.05).

As IL-6 did not change c-Met expression in ANBL-6 (Fig. 3C), we decided to further examine the intracellular pathways involved in potentiation of IL-6-induced proliferation by c-Met in this cell line. Cells were starved for 4 h to increase endogenous HGF levels. PHA-665752 reduced the modest phosphorylation of p44/42-MAPK in the control wells (Fig. 5A, top panel, lanes 1 and 2), indicating that the autocrine HGF activated p44/42-MAPK weakly. Adding IL-6 increased p44/42-MAPK phosphorylation substantially. When cells were treated with the c-Met tyrosine kinase inhibitor PHA-665752 there was almost complete abrogation of IL-6-induced phosphorylation of p44/42 MAPK (lane 4 vs. lane 3). Similarly, the antibody blocking HGF binding to c-Met inhibited IL-6 induced p44/42 MAPK phosphorylation (lane 5 vs. lane 3) in a similar manner as PHA-665752. Taken together, the results indicate that IL-6 was dependent on c-Met signaling for full activation of p44/42 MAPK. In contrast, IL-6-induced phosphorylation of STAT3 (Fig. 5A, middle panel) was independent of the c-Met inhibitor PHA-665752 and the antibody inhibiting HGF binding to c-Met.

The p44/42 MAPK are downstream targets of active Ras. As seen in Fig. 5B, Ras activation by IL-6 was also dependent on c-Met signaling as PHA-665752 reduced the effect of IL-6 substantially. Thus, the dependency on c-Met in IL-6-mediated p44/42 MAPK activation is a consequence of dependency on c-Met in IL-6-mediated Ras activation. Taken together, the results suggest that the basis for the potentiating role of c-Met signaling on IL-6-induced proliferation is upstream of Ras.

In analogy with previous reports (30), we found that the Ras-MAPK pathway was important for proliferation of ANBL-6 cells because the MEK1/2-inhibitors PD98059 and U126 both inhibited proliferation in these cells (Fig. 5C).

### IL-6 was dependent on c-Met for phosphorylation of Gab-1 and Shp2

**Figure 6:**
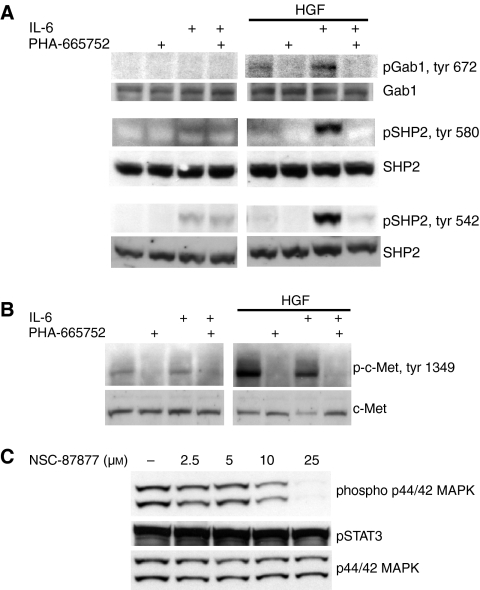
HGF was necessary for Gab-1-Shp2 activation in ANBL-6 cells. (A) ANBL-6 cells were incubated overnight with a 1 : 750 dilution of anti-HGF serum (to inhibit autocrine HGF-stimulation and subsequently increase c-Met expression), then washed, cultured with or without 200 nm PHA-665752 for 15 min and stimulated for 10 min with or without HGF (100 ng/mL) or IL-6 (1 ng/mL). Lysates were prepared and subjected to Western blot analysis. Membranes were probed with antibodies against phospho-Gab1 tyr 627, phospho-Shp2 tyr 580 or 542. After stripping of membranes, probing with antibodies against total Gab-1 or total Shp2 were used to control gel loading. (B) Cells were treated as in (A). Immunoblots were probed with an antibody detecting phosphorylated tyrosine 1349 of c-Met. After stripping of the membrane, total c-Met level was detected with a c-Met-specific antibody. (C) ANBL-6 cells were preincubated with different concentrations of the Shp2-inhibitor NSC-87877 for 5 h and then stimulated with 1 ng/mL IL-6 for 10 min. Immunoblots were probed with antibodies against phospho-p44/42 MAPK or phospho-STAT3 tyr 705, stripped and reprobed with an antibody against total p44/42 MAPK.

The results above indicated that molecules upstream of Ras are possible mediators of the synergy between HGF and IL-6 in inducing proliferation in ANBL-6 cells. Among candidate molecules in this pathway are the tyrosine phosphatase Shp2 and the adaptor molecule Gab-1 (31). In Fig. 6A,B, we examined the ability of HGF and IL-6 to induce phosphorylation of Gab1 and Shp2 in ANBL-6 cells. Because these cells produce HGF endogenously resulting in low c-Met expression, we preincubated the cells over night with anti-HGF serum to increase c-Met expression before addition of IL-6 for 10 min with or without the presence of the c-Met kinase inhibitor as indicated in Fig. 6A,B. IL-6 induced low phosphorylation of tyrosine 542 on Shp2 under these conditions (Fig. 6A left hand panels). In contrast, HGF induced low but detectable phosphorylation of Gab1. Importantly, in the presence of HGF, the phosphorylation of Shp2 was further increased with IL-6 (Fig. 6A right hand panels). Furthermore, the Gab1 and Shp2 phosphorylation induced with the combination of HGF and IL-6 was markedly reduced in the presence of the c-Met kinase inhibitor. These results indicate that the combination of HGF and IL-6 gave more pronounced activation of Shp2 than either cytokine alone, suggesting that Shp2 activation induced by IL-6 also is dependent on c-Met activation.

IL-6 has been reported to phosphorylate the IGF-1 receptor as basis for synergy between IL-6 and IGF-1 (32). Phosphorylation of c-Met induced by IL-6 could have been an explanation for potentiation of Shp2 phosphorylation in ANBL-6 cells. However, this seemed not to be the case (Fig. 6B).

To see if Shp2 activation was involved in activation of p44/42 MAPK activation, we tested the effect of the novel Shp2-inhibitor NSC-87877. This inhibitor binds to the catalytic cleft of Shp2 and inhibits both basal, and EGF-induced Shp2 phosphatase activity as well as EGF-induced p44/42 MAPK phosphorylation which is known to be dependent on Shp2 (33). In the presence of IL-6 and endogenous HGF, NSC-87877 inhibited phosphorylation of p44/42 MAPK in ANBL-6 cells in a dose-dependent manner, without affecting the phosphorylation of STAT3 (Fig. 6C). These results suggest that whereas Shp2 is involved in p44/42 MAPK activation, it has no role in STAT3 phosphorylation which is entirely dependent on IL-6 in this setting. Furthermore, the synergy observed in Ras-MAPK signaling is dependent on the synergy in phosphatase activity of Shp2.

## Discussion

The main finding reported here is that IL-6-induced proliferation may be dependent on c-Met signaling in myeloma cells. The potentiating effect of HGF/c-Met on IL-6 signaling could be explained by two mechanisms: (i) IL-6 increased the level of c-Met on the cell surface of myeloma cells making cells more sensitive to HGF; and (ii) IL-6 relied on HGF/c-Met to fully activate the Ras-MAPK pathway possibly through Shp2 activation.

HGF is found in bone marrow plasma of both healthy subjects and myeloma patients (5), and bone marrow stromal cells constitutively produce HGF (34). Moreover, syndecan-1 binds HGF on the surface of myeloma cells (5) bringing HGF in close proximity of its receptor c-Met. Immunohistochemical staining for HGF on bone marrow biopsies revealed that plasma cells from almost all myeloma patients stained positive for HGF (K. W. Wader, unpublished data). In this context, the IL-6-induced increase in c-Met expression as shown here may become vital for HGF sensitivity and growth promotion of the cells. This is in line with other reports indicating that increase of c-Met expression enhances both the biologic effects of HGF and c-Met signaling in various cell types (35–37). A recent publication also indicates that the level of c-Met expression is important for the survival of myeloma cells as partly downregulation of c-Met lead to myeloma cell death (38). Moreover, *in vivo* induction of the IGF-1 receptor has been reported in the murine myeloma model 5T33MM, and this induction was necessary for biological effects of IGF-1 in these experiments (39).

Inhibiting c-Met had substantial effects on IL-6-induced proliferation in four out of nine primary samples, although the frequency of this mechanism in primary myeloma patients is hard to estimate due to the low numbers of samples. These results are intriguing in the light of the work of Chng et al. (9). They describe a cluster of hyperdiploid patients with high expression of HGF and IL-6 suggesting biologic importance of these cytokines in these patients. As part our routine check on MM patients, we screen for the genetic aberrations denoted in Table 1. These data are not sufficient to designate patients to the hyperdiploid group or even less to the HGF/IL-6 subgroup of hyperdiploid myeloma. Nevertheless, response to c-Met inhibition was present in patients with t(6; 14) or t(11; 14) or without IgH translocations. This suggests response in non-hyperdiploid (and thus not exclusively in hyperdiploid) cases because IgH translocations are strongly associated with non-hyperdiploid myeloma and a rare event in hyperdiploid patients (40, 41). Further studies are necessary to see, if hyperdiploid patients with high HGF- and IL-6-expression are subjected to synergy between IL-6 and HGF, and if they can benefit from c-Met inhibition.

The potentiating effect of c-Met signaling in IL-6-induced p44/42 MAPK activation in ANBL-6 cells was intriguing and a novel observation. Neither HGF nor IL-6 alone could induce Ras-MAPK signaling, but the combination of HGF and IL-6 was necessary to activate this pathway. The Ras-MAPK pathway is a major regulator of cell proliferation, and has previously been shown to be important for myeloma cell proliferation *in vitro* and *in vivo* (30). However, the role of c-Met as a regulator of IL-6-induced Ras-MAPK signaling has to our knowledge not been shown in myeloma cells before.

The synergy between IL-6 and c-Met in ANBL-6 cells was also evident at the level of Shp2 phosphorylation. Thus, the synergy between IL-6 and HGF must converge on Shp2 or be a result of synergy upstream of Shp2. IL-6 did not induce phosphorylation of c-Met or Gab1 as HGF did while IL-6 treatment resulted in phosphorylation of Shp2. Thus, there may be two ways in which Shp2 can be phosphorylated: IL-6 may induce Shp2 phosphorylation on tyrosine 542 whereas c-Met signaling potentiates the phosphorylation of both tyrosine 542 and 580 in a process dependent on Gab1. There is some support for such a mechanism in the literature as it has been shown that Shp2 can directly bind to the cytoplasmic tail of gp130 and become activated (42). Furthermore, IL-6 has previously also been shown to phosphorylate Shp2 in the myeloma cell line MM1.S (43). There is also evidence that the double phosphorylation of Shp2 on tyrosines 542 and 580 is important for full catalytic activity of Shp2 (44). The results presented here indicate that both IL-6 and c-Met activation may be required for full catalytic activity of Shp2.

Shp2 activation appeared to be necessary for the activation of p44/42 MAPK as the novel SHP2 inhibitor NSC-87877 abrogated cytokine-mediated MAPK phosphorylation in ANBL-6. NSC-87877 is also known to inhibit the tyrosine phosphatase Shp1; however, Shp1 has been shown to negatively control receptor signaling (45), and even to reduce MAPK-activation in thyroid carcinoma and neurons (46, 47).

Here, we show that c-Met signaling may be important in myeloma cell proliferation induced by IL-6. Targeting HGF/c-Met may therefore attenuate growth promotion by other growth factors than HGF, and c-Met signaling may be a target for therapy also in multiple myeloma.

## Supporting Information

**Table S1.** Probes for FISH.
